# Engineering T cell therapies for lung cancer

**DOI:** 10.1016/j.omton.2026.201172

**Published:** 2026-03-18

**Authors:** Hyatt Balke-Want, Vimal Keerthi, Steven A. Feldman

**Affiliations:** 1 Department I of Internal Medicine, Center for Integrated Oncology Aachen Bonn Cologne Duesseldorf, University of Cologne, Medical Faculty and University Hospital Cologne, Cologne, Germany; 2 Cancer Center Cologne Essen (CCCE), Cologne, Germany; 3 Center for Molecular Medicine Cologne (CMMC), University of Cologne, Cologne, Germany; 4 Center for Cancer Cell Therapy, Stanford Cancer Institute, Stanford University, Stanford, CA, USA; 5 Laboratory for Cell and Gene Medicine, Stanford Medicine, Stanford University, Stanford, CA, USA; 6 Division of Blood and Marrow Transplantation, Stanford Medicine, Stanford University, Stanford, CA, USA

## Main text

In our recent publication, “c-JUN enhances CRISPR knockin anti-B7-H3 CAR T cell function in small cell lung cancer and thoracic SMARCA4-deficient undifferentiated tumors,”^1^ we utilized a non-viral CRISPR-Cas9 knockin (CKI) platform to engineer CAR T cells targeting small cell lung cancer (SCLC) and thoracic SMARCA4-deficient undifferentiated tumors (thoracic SMARCA4-deficient UTs). Lung cancer remains the leading cause of cancer death. Viral vector production for T cell engineering poses a major bottleneck for both developing novel engineered T cells with synthetic immunoreceptors and their broad application. We posit that non-viral engineering via CKI can overcome these limitations and enable broad access to engineered T cell therapies, including CAR T cells, against lung cancer.^2^

In our work, we focused on SCLC for two key reasons. First, its highly aggressive clinical course and limited therapeutic advances over the last decades highlight an unmet need. Second, developing engineered T cell therapies against solid tumors suffers from biological drawbacks stemming from the suppressive tumor microenvironment (TME) and T cell exhaustion due to high tumor burden. However, in SCLC, frontline platinum-based chemotherapy results in high remission rates, albeit not durable, thus providing a unique opportunity to consolidate initial treatment success with CAR T cells and thereby potentially create durable benefit for patients with SCLC.^3^

To enhance CAR T cell function against immunosuppressive SCLC tumors using existing CAR formats (e.g., DLL3-targeting CARs), we initially aimed to characterize the expression of potentially immunosuppressive ligands. Surprisingly, we found CD276 (encoding B7-H3), which serves as a CAR T cell target in other cancer histologies, to be the most upregulated gene. Expression of *CD276*/B7-H3 is prevalent across SCLC cases and our cell line panel, including lines recently reclassified from SCLC to thoracic SMARCA4-deficient UTs. Notably, in the MYC-driven autochthonous RPM (Rb1fl/flTrp53fl/flMycLSL/LSL) SCLC mouse model, *Cd276* expression remained consistently expressed throughout disease progression, even as tumors transitioned from neuroendocrine to non-neuroendocrine states and lost other key targets (*Dll3* and *Sez6*). Importantly, the non-neuroendocrine SCLC subtype is linked to therapy resistance and therefore might cause treatment failure.^4^ While DLL3-targeted CAR T cell trials are underway in SCLC,^5^ B7-H3 has only been tested via antibody drug conjugates (ADCs) in SCLC, which lack immunological memory formation and clinically yielded modest durability (median DOR = 5.7 months), despite strong initial responses (ORR = 63.9%).^6^

Although SCLC cell lines responded robustly to anti-B7-H3 CAR T cells, the thoracic SMARCA4-deficient UT cell line H841, despite high B7-H3 levels, resisted complete clearance *in vitro* and *in vivo*. Further analysis revealed elevated TGF-β secretion by H841 cells, consistent with prior reports of TGF-β suppressing CAR T cytokine production and activity.^7^ We hypothesized that co-expressing the AP-1 transcription factor c-JUN with our B7-H3 CAR would counteract TGF-β-mediated inhibition, building on prior evidence that c-JUN confers exhaustion resistance and enhances T cell efficacy in solid tumors.^8^ Thus, we next aimed to characterize the effects of c-JUN in our CKI B7-H3 CAR T against SCLC and thoracic SMARCA4-deficient UT and further delineate the mode of action in our experimental system. In line with previous work, when we co-inserted c-JUN, we observed boosted activity of not only CKI B7-H3 CAR T cells against antigen low SCLC but also TGF-β-expressing H841 cells. Our in-depth characterization revealed that c-JUN drove IL-2 and IFN-γ as well as type-II cytokine expression (including IL-4, IL-13, and IL-31) in CD8^+^ CKI B7-H3 CAR T cells. Interestingly, the expression of type-II cytokines has previously been shown to associate with ultra-long-term remission in B-ALL treated with CAR T cells^9^ and thus provides a mechanistic explanation for the enhanced activity of B7-H3 CAR T cells against thoracic SMARCA4-deficient UT. Additionally, c-JUN co-insertion reduced exhaustion by downregulating NR4A2, a key exhaustion-associated transcription factor.

To facilitate clinical translation, we implemented production of B7-H3 CAR T cells, including c-JUN co-expressing iterations, using our non-viral CRISPR-Cas9 platform. To this end, we optimized our engineering parameters and electroporation conditions with nanoplasmid DNA templates, which are available at good manufacturing practice (GMP) grade. Our construct design employed homology directed repair at the TRAC locus following CRISPR-Cas9-induced double-strand breaks, introducing a short EF-1α promoter (EFS), the B7-H3 CAR with or without c-JUN, and a mutant dihydrofolate reductase (DHFR-FS) conferring resistance to the clinically used agent methotrexate (MTX), thereby enabling enrichment of successfully edited CAR T cells. Building on our prior optimization of MTX dose, initiation time point, and treatment duration,^10^ we successfully adapted this strategy to manufacture c-JUN+B7-H3 CAR T cells at a clinical scale within a 10–14 day process. Finally, we have provided evidence that our non-viral CAR T cell products engineered with CRISPR-Cas9 do not show any signs of genotoxicity. Our sgRNA has already been extensively characterized by us previously^10^ and did not show any signs for off-target editing, nor did we detect off-target insertion. Via ddPCR, we were also able to estimate that CRISPR knockin occurred in a mono-allelic manner given the on-target copy number of one.

In summary, we have provided a framework to generate non-viral CKI CAR T cells in a broadly accessible manner and specifically tested non-viral CKI B7-H3 CAR T cells in SCLC and thoracic SMARCA4-deficient UT. Our approach efficiently integrates even large tricistronic constructs (>3.6 kb, including c-JUN) to enhance B7-H3 CAR T function at clinical scale, with no evidence of genotoxicity. Furthermore, our platform allows us to seamlessly integrate additional CRISPR-Cas9-based modifications to optimize CAR T cell performance. Given the consistent expression of B7-H3 throughout SCLC progression and across patients, as well as the recent FDA approval of the bispecific T cell engager tarlatamab for SCLC, which targets the neuroendocrine marker DLL3, CAR T cells targeting B7-H3 warrant further clinical investigation. Future consolidative strategies post frontline platinum-based chemotherapy promise key insights for SCLC and solid tumors broadly.

## Declaration of interests

H.B.-W. received research funding from Wilson Wolf Manufacturing, LLC. S.A.F. serves on the scientific advisory boards for MFX, Autolomous, and Advanced Cell Therapy Centre (Oslo University Hospital); serves as a board member for Biotech Partners and Act for Hope; and has patents in the field of cell therapy and receives royalties from NIH related to those patents.
